# The analgesic effects of triptolide in the bone cancer pain rats via inhibiting the upregulation of HDACs in spinal glial cells

**DOI:** 10.1186/s12974-017-0988-1

**Published:** 2017-11-02

**Authors:** Xiao-Fan Hu, Xiao-Tao He, Kai-Xiang Zhou, Chen Zhang, Wen-Jun Zhao, Ting Zhang, Jin-Lian Li, Jian-Ping Deng, Yu-Lin Dong

**Affiliations:** 1 Department of Human Anatomy & K.K. Leung Brain Research Centre, Preclinical School of Medicine, The Fourth Military Medical University, Xi’an, 710032 China; 2 Department of Orthopedics, Xijing Hospital, The Fourth Military Medical University, Xi’an, 710032 China; 3 State Key Laboratory of Military Stomatology and National Clinical Research Center for Oral Disease, Department of Periodontology, School of Stomatology, The Fourth Military Medical University, Xi’an, 710032 China; 4 Department of Neurosurgery, Tangdu Hospital, The Fourth Military Medical University, Xi’an, 710038 China; 5 Student Brigade, The Fourth Military Medical University, Xi’an, 710032 China

**Keywords:** Bone cancer pain, Triptolide, Antinociceptive effect, Glial cell, Histone deacetylase

## Abstract

**Background:**

Bone cancer pain (BCP) severely compromises the quality of life, while current treatments are still unsatisfactory. Here, we tested the antinociceptive effects of triptolide (T10), a substance with considerable anti-tumor efficacies on BCP, and investigated the underlying mechanisms targeting the spinal dorsal horn (SDH).

**Methods:**

Intratibial inoculation of Walker 256 mammary gland carcinoma cells was used to establish a BCP model in rats. T10 was intrathecally injected, and mechanical allodynia was tested by measuring the paw withdrawal thresholds (PWTs). In mechanism study, the activation of microglia, astrocytes, and the mitogen-activated protein kinase (MAPK) pathways in the SDH were evaluated by immunofluorescence staining or Western blot analysis of Iba-1, GFAP, p-ERK, p-p38, and p-JNK. The expression and cellular localization of histone deacetylases (HDACs) 1 and 2 were also detected to investigate molecular mechanism.

**Results:**

Intrathecal injection of T10 inhibited the bone cancer-induced mechanical allodynia with an ED_50_ of 5.874 μg/kg. This effect was still observed 6 days after drug withdrawal. Bone cancer caused significantly increased expression of HDAC1 in spinal microglia and neurons, with HDAC2 markedly increased in spinal astrocytes, which were accompanied by the upregulation of MAPK pathways and the activation of microglia and astrocytes in the SDH. T10 reversed the increase of HDACs, especially those in glial cells, and inhibited the glial activation.

**Conclusions:**

Our results suggest that the upregulation of HDACs contributes to the pathological activation of spinal glial cells and the chronic pain caused by bone cancer, while T10 help to relieve BCP possibly via inhibiting the upregulation of HDACs in the glial cells in the SDH and then blocking the neuroinflammation induced by glial activation.

## Background

The most common cancer pain arises from bone metastasis which is observed in nearly 70% of patients who died of cancer [1, 2]. Bone cancer pain (BCP) is present in around one third of patients with bone metastasis and seriously impacts the quality of life [2, 3]. With the rapid increase of the incidence rates, survival rates and surviving time of cancer in modern society, strategies to manage BCP have become increasingly important [2]. Currently, BCP is largely managed with an “analgesic ladder” originally promulgated by the World Health Organization in 1986 which mainly contains opioids and nonsteroidal anti-inflammatory drugs (NSAIDs) [2]. Other adjuvant therapies, including radiation therapy, surgery, chemotherapy, and antiepileptics, are also used to control cancer pain [2]. However, current therapies have been unsatisfactory because of the relative ineffectiveness and the severe side effects, such as the tolerance, withdrawal, dependence, and addiction of opioids [3]. Studies have shown that at least 20–40% of cancer pain is not adequately relieved and BCP has been considered one of the most difficult pain conditions to treat [2, 4]. Thus, it is time to move on to develop new analgesic therapies that are efficacious and/or could reduce the use of current therapies, when utilized in combination, thus abate their side effects.

Triptolide (T10) is a major active ingredient extracted from the Chinese herb, *Tripterygium wilfordii* hook F (TWHF) [5]. It has been shown that T10 can effectively inhibit many kinds of cancers [6], including osteosarcoma [7, 8], pancreatic cancer [9, 10], breast cancer [11, 12], and leukemia [13, 14]. Recent studies have demonstrated T10 to be effective in ameliorating some non-cancer pain status, such as neuropathic pain [5, 15, 16]. However, the effects of T10 on cancer pain remains elusive. Recently, Hang et al. have reported that intrathecal T10 could relieve the BCP in rats [17]. However, the mechanisms of the analgesic effects of T10 are still unclear, especially in BCP.

In terms of neuropathic and inflammatory pains, it has been proposed that the activated microglia and astrocytes release proinflammatory cytokines, establishing a neuroinflammatory framework and modulating pain processing [18, 19]. Collective evidence also supports the implication of glial over-activation and the subsequent neuroinflammation in the spinal cord in the emergence and development of BCP [20–23]. However, increasing studies have shown that BCP is a pain with mixed mechanism, which is different from non-cancer pain states [3]. Therefore, although T10 exerts considerable immunoregulation activity in its clinical use for the treatment of autoimmune diseases [5] and the anti-inflammatory effects in the spinal dorsal horn (SDH) have been shown to be an important mechanism for its analgesic effects on neuropathic pain [15, 16], it is still unclear whether and how T10 helps to relieve BCP through its immunoregulation actions on glial cells.

In the past years, epigenetic mechanisms in the central nervous system (CNS), including DNA methylation, histone modifications, and miRNA activity, have been reported to drive long-lasting molecular and cellular changes in chronic pain conditions [24]. New results in our lab about the epigenetic modifications in BCP showed that the upregulation of class I histone deacetylases (HDACs) in the SDH plays a critical role in the neuroinflammation response and chronic pain in the rat BCP model (in submission). On the other hand, studies about the anti-tumor effects of T10 have demonstrated that T10 could regulate histone modifications by altering molecules like histone methyltransferases and demethylases [25, 26], suggesting the epigenetic modulation effects of T10. Therefore, we hypothesized that T10 might influence the bone cancer-induced activation of spinal glial cells via epigenetic mechanisms.

Thus, the aims of the present study were to investigate (1) the analgesic effects of T10 on BCP; (2) whether bone cancer induces activation of spinal glial cells and T10 helps to alleviate BCP via modulating glial activation in the SDH; and (3) the molecular mechanisms underlying the effects of T10 by targeting HDACs.

## Methods

### Animals

Female Sprague-Dawley rats (200–220 g) were housed in a temperature-controlled room with free access to food and water at 22–25 °C on a 12-h light/dark cycle. All of the animal study protocols were conducted with strict adherence to the experimental protocol of the Animal Use and Care Committee for Research and Education of the Fourth Military Medical University (Xi’an, China, Animal Ethical Committee approval number: FMMU-AEEA-20151106) and the IASP’s guidelines for pain research [27].

### Intrathecal implantation

Intrathecal implantation was performed as described in our previous studies [16, 28] by inserting polyethylene tubing through which the drug was directly injected into the subarachnoid space of the lumbar enlargement. After surgery, neurologically normal rats were injected with 2% lidocaine (10 μL) through the intrathecal catheter to confirm that the polyethylene tubing was in the subarachnoid space. Only those rats showing complete paralysis of both hind limbs after the administration of lidocaine were used for the subsequent experiments. At the end of each experiment, the position of the polyethylene tubing in the intrathecal space at the lumbar enlargement was visually verified by exposing the lumbar spinal cord. Data from rats with incorrect PE tubing position were discarded from the study.

### Cancer cell preparation

The Walker 256 rat mammary gland carcinoma cell line was purchased from American Type Culture Collection (ATCC, USA). To obtain the cancer cells used in the BCP model, as described previously [1], 0.5 mL (2 × 10^7^ cells/mL) of cancer cells was injected into the abdominal cavities of adult female SD rats. After 7–10 days, 2 mL of ascitic fluid was extracted and centrifuged for 3 min at 1500 rpm. The pellet was washed with 10 mL of phosphate-buffered saline (PBS, pH = 7.4) and centrifuged again for 3 min at 1500 rpm. After being resuspended in 1 mL PBS, the cells were counted with a hemocytometer, diluted to achieve a final concentration of 5 × 10^5^ cells/10 μL PBS and kept on ice until injection. The same final concentration of cells were boiled for 20 min and then used in sham group.

### Bone cancer pain model

BCP model was conducted as previously described [1]. Briefly, following complete anesthesia with sodium pentobarbital (i.p. 50 mg/kg), a 1-cm incision was made on the right leg of the rat to expose the medial aspect of the tibia. Ten microliters of Walker 256 carcinoma cells (5 × 10^5^ cells) was slowly injected into the intramedullary cavity of the tibia. The syringe was then removed, and the injection site was immediately closed with bone wax. Then, penicillin was applied to the wound. In the sham group, the same procedures were followed, except that an equal volume of heat-treated carcinoma cells was administered instead of normal carcinoma cells.

### Drug administration

**Fig. 1:**
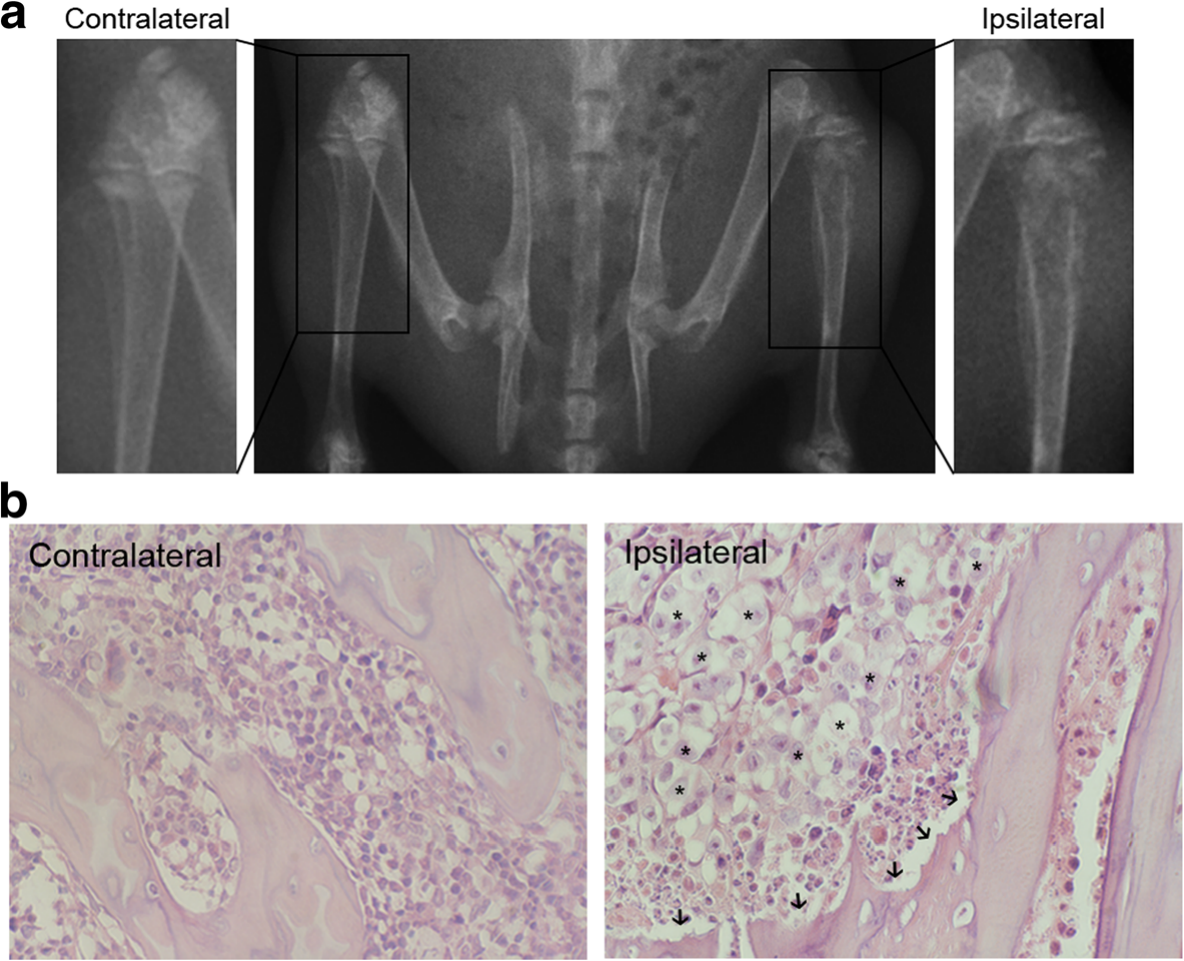
Tibia destruction 14 days after Walker 256 cells inoculation. **a** Radiographs of the contralateral and ipsilateral tibia show the bone destruction in the tibia inoculated with cancer cells. **b** Histological images of tibia sections stained by hematoxylin-eosin. The arrows and asterisks indicate the bone destruction on trabecular surface and the cancer cells in bone marrow respectively

Triptolide (T10, Sigma, St. Louis, MO) was dissolved in dimethylsulfoxide (DMSO, Sigma-Aldrich) and then diluted in saline to a concentration of 1 μg/μL, with the final concentration of DMSO as 0.4%. The doses for the intrathecal administration of T10 were selected according to previous studies [16, 17] and our preliminary experiments. Different doses (1.5, 3, 12, 48, and 96 μg/kg) of T10 were intrathecally (i.t.) injected once per day for 7 days from postoperative days (POD) 7 to 13 (Fig. 1a). The animals were divided into six groups: sham group (*n* = 8), BCP group (*n* = 8), sham + vehicle group (*n* = 8), BCP + vehicle group (*n* = 8), BCP + T10 group [*n* = 40, 8 for each of the 5 subgroups (1.5, 3, 12, 48 or 96 μg/kg for each subgroup)], and sham + T10 group (*n* = 8, 12 μg/kg T10 was injected into sham-operated rats). A behavioral test, namely, mechanical allodynia test, was conducted on 1 and 5 days before BCP modeling surgery to get baseline data and was also performed on 3, 5, 7, 9, 11, 13, 15, 17, and 19 days after modeling to assess the chronic pain in different groups. For immunofluorescent detection and Western blot analysis on POD 14, T10 was administrated at the same time points (Fig. 1a), while the dose of T10, 5 μg/kg, was chosen according to the ED_50_ of T10 which was calculated from the behavioral data.

### Mechanical allodynia test

Mechanical allodynia was tested with von Frey filaments according to our previous reports [15, 28]. Before baseline testing, the rats were habituated to the testing environment for 5 days. Baseline data were tested both before and after the intrathecal tube implantation. Animals that showed obviously different data between these two tests were discarded. For the remaining animals internalized in the subsequent studies, the average of these two baseline tests was recorded as a baseline data. In the test, rats were placed under individual inverted plastic boxes on an elevated mesh floor and allowed to acclimate for 30 min before testing. Paw withdrawal thresholds (PWTs) were measured with von Frey filaments (Stoelting, Kiel, WI, USA) in a blinded manner. The ipsilateral hind paw was pressed with one of a series of von Frey filaments with increasing stiffness (1, 2, 4, 6, 8, 10, 15, and 26 g) applied to the plantar surface for 5–6 s for each filament. Rapid pulling back, biting, licking, or shaking of the ipsilateral hind limb was considered a positive sign of a withdrawal response. Applications were separated by 5-min intervals to allow the animal to cease possible responses and return to a relatively inactive position. For each trial, the same hind paw was stimulated 10 times by a single von Frey filament before being stimulated by the next larger filament. The minimal value causing at least six responses was recorded as the PWTs. The area under the time-course curve (AUC) values was used to measure the summed effects of different treatment and calculated in the time from the first day of drug administration to the final tests, i.e., day 7 to day 19. In the calculation of antinociception percentage, the AUC of every animal was used. The AUCs of rats in sham + veh group and BCP + veh group were used to get two intra-group mean values: AUC_sham_ and AUC_BCP_. Then, the AUC of a certain animal in the T10-treated groups (AUC_i_) was used to calculated the percentage of antinociception for that rat with the following formula: antinociception (%) = (AUC_i_ − AUC_BCP_)/(AUC_sham_ − AUC_BCP_) × 100. After this, the intra-group mean values of antinociception percentage in BCP + T10 groups were calculated, as shown in Fig. 3c–e.

### Western blot analysis

Fourteen days after surgery, the rats were anesthetized and rapidly sacrificed. The ipsilateral L5 spinal cord segment was dissected on dry ice and then homogenized in SDS sample buffer (10 mL/mg tissue) with a mixture of proteinase and phosphatase inhibitors (Sigma). The protein concentrations were estimated by the bicinchoninic acid (BCA) method. The samples were then processed by Western blot analysis with primary antibodies: goat anti-ionized calcium-binding adaptor molecule 1 IgG (ionized calcium-binding adaptor molecule 1 (Iba-1), a marker of microglia, 1:1000, Abcam, Cambridge, MA, USA), mouse anti-glial fibrillary acidic protein (GFAP, a marker of astrocytes, 1:5000, Chemicon, Temecula, CA, USA), rabbit-anti p-extracellular signal-regulated kinase (p-ERK; Cell Signaling, Danvers, MA, USA), p-p38 (1:1000; Cell Signaling) and p-Jun N-terminal kinases (p-JNK, 1:1000; Cell Signaling), rabbit anti-HDAC1 (1:1000; Sigma), and rabbit anti-HDAC2 (1:1000; Cell Signaling). The levels of target proteins were normalized against those of β-actin and expressed as fold changes relative to the sham-vehicle group.

### Immunofluorescent histochemistry

Fourteen days after surgery, the rats were anesthetized and transcardially perfused with 100 mL of 5 mM PBS (pH 7.4), followed by 500 mL of 4% (*w*/*v*) paraformaldehyde in 0.1 M phosphate buffer (PB, pH 7.3). The L5 spinal cord segment was removed and immersed in 30% (*w*/*v*) sucrose in 0.1 M PB overnight at 4 °C. Transverse frozen spinal sections (25 μm) were cut in a cryostat (Leica CM1800; Heidelberg, Germany). All sections were serially collected in 0.01 M PBS (pH 7.4) and used for immunofluorescent histochemical staining.

After being rinsed in 0.01 M PBS three times (10 min each) and blocked in 0.01 M PBS containing 10% normal donkey serum and 0.3% Triton X-100 for 1 h at room temperature (RT), the sections were incubated for 1 h at RT and then overnight at 4 °C with primary antibodies, goat anti-Iba-1 (1:800; Abcam), mouse anti-GFAP (1:5000; Chemicon), mouse anti-NeuN (1:2000; Chemicon), rabbit anti-HDAC1 (1:200; Sigma-Aldrich), and rabbit anti-HDAC2 (1:400; Cell Signaling), in PBS containing 0.3% (*v*/*v*) Triton X-100, 0.25% (*w*/*v*) λ-carrageenan, and 5% (v/v) donkey serum (PBS-XCD). After being washed three times in 0.01 M PBS (10 min each), sections were incubated for 6 h at RT with donkey secondary antibodies to corresponding species conjugated with Alexa 488 or Alexa 594 (all 1:500; Millipore, Billerica, MA, USA). Finally, the sections were mounted onto clean glass slides, air-dried, and coverslipped. The sections were observed under a confocal laser scanning microscope (FV-1000, Olympus, Tokyo, Japan) with the appropriate laser beams and filter settings for Alexa 488 (excitation, 488 nm; emission, 510–530 nm) and Alexa 594 (excitation, 543 nm; emission, 590–615 nm). Confocal images were obtained, and digital images were captured and analyzed using a Fluoview 1000 (Olympus). The specificity of the immunofluorescent staining was tested by omitting the specific primary antibodies. No immunoreactive products were detected (data not shown).

Quantitative analysis of the percentage of immunostaining surface in the spinal cord laminae I–II and the whole SDH was conducted with Image Pro-Plus program as described previously [29]. Briefly, the background in pictures was first subtracted with a uniform standard. The regions for laminae I–II and the whole SDH in the spinal sections were artificially selected. Then, the threshold values of fluorescent intensity for positive immunoreactivity were set and the percentage of immunostaining areas were obtained by the Image Pro-Plus program 6.0. For each animal, the data from five different rostrocaudal planes within L4 and L5 spinal cord segments was obtained and four animals in each group were evaluated to get the mean values.

### Dose-effect curve and ED_50_ calculation

The T10 dosages were transformed into logarithm dose with GraphPad Prism software, and the nonline fit was performed to build the log(dose)-effect curve [16]. Based on the log(dose)-effect curve, the ED_50_ of the analgesic effects of T10 was calculated by the GraphPad Prism version 5.01 for Windows (San Diego, CA, USA, http://www.graphpad.com/).

### Statistical analyses

All data were analyzed by researchers blinded to the surgery and reagents used. Analysis of the pain behavior test conducted at different time points was performed by a two-way analysis of variance (ANOVA) followed by Bonferroni’s multiple comparison test at each time point. The data from the Western blot analysis and immunofluorescent staining were analyzed by one-way ANOVA with Student-Newman-Keuls (SNK) post hoc test. All data were analyzed by GraphPad Prism version 5.01 and presented as mean ± SEM. *p* < 0.05 was considered statistically significant.

## Results

### Evaluation of the bone destruction in BCP model

To verify the validation of the BCP model, radiological imaging and histological analysis of the rat tibia were conducted to assess the bone destruction 14 days after cancer cell inoculation. In radiographs (Fig. 1a), compared with the contralateral tibia of BCP rats without cancer cell injection, the tibia inoculated with Walker 256 cells (ipsilateral) showed abnormal radiographical structures of both cortical bone and marrow cavity in the proximal tibia, indicating the bone destruction around the cancer cell injection site. Hematoxylin-eosin staining of tibia sections showed no cancer cells or bone destruction in the contralateral tibia of BCP model rats (Fig. 1b). The ipsilateral tibia, however, showed obvious cancer cell growth in marrow cavity and bone destruction along the surfaces of trabecular and cortical bone, indicating the development of bone cancer in the tibia.

### Cancer cell inoculation in tibia induced bilateral mechanical allodynia of hind paws

**Fig. 2:**
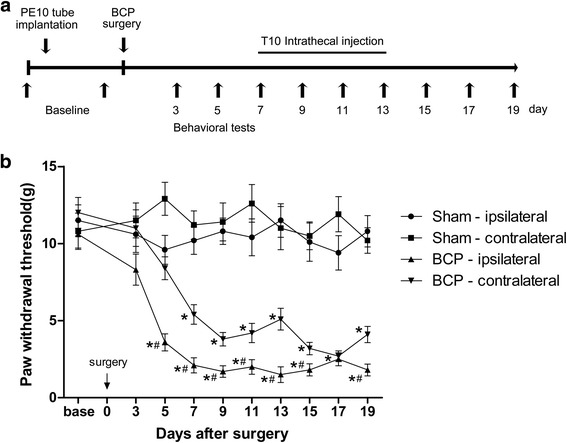
Bone cancer in tibia induced mechanical allodynia of hindpaw. **a** Experimental paradigms. **b** Bone cancer resulted in prominent decrease in the PWTs of ipsilateral hind paw on POD 5 to 19 and also of the contralateral hind paw on POD 7 to 19. *n* = 8 per group, *p < 0.05 vs. sham-ipsilateral; ^#^ *p* < 0.05 vs. BCP-contralateral

To assess the chronic pain status induced by BCP model, the mechanical allodynia of animal hind paws were evaluated (Fig. 2a). In sham rats, the contralateral and ipsilateral hind paws showed no difference in PWTs at all the time points (Fig. 2b). However, the PWTs of BCP-ipsilateral hind paws were significantly lower than those of both sham-ipsilateral on POD 5 to 19 and also lower than those of BCP-contralateral at most time points after POD 5, indicating the bone cancer-induced mechanical allodynia of hind paws. The PWTs of BCP-contralateral hind paws were also significantly lower than those of sham-ipsilateral on POD 5 to 19, although the thresholds were still higher than those of BCP-ipsilateral.

### Intrathecal T10 administration attenuated the bone cancer-induced mechanical allodynia in a dose-dependent manner

**Fig. 3:**
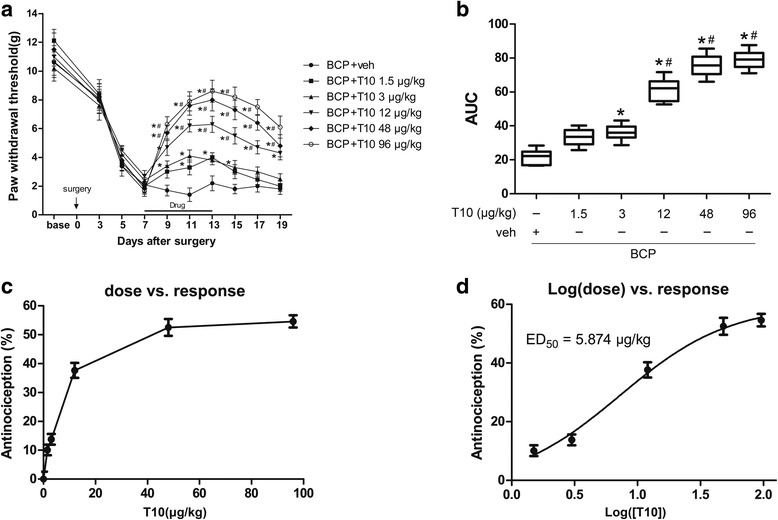
Effects of intrathecal T10 administration on bone cancer induced mechanical allodynia. **a** Intrathecal injection of T10 from day 7 to day 13 did not affect the PWTs of the sham-operated group. However, T10 dose-dependently alleviated the mechanical allodynia in the BCP rats. This effect was still observed 6 days after drug withdrawal at day 19. **b** The AUCs for different groups were calculated to perform statistical analysis. The dose-effect or log (dose)-effect curves for the analgesic effects of intrathecal T10 were shown in **c** and **d**. The ED_50_ of T10 on bone cancer-induced mechanical allodynia was 5.874 μg/kg. *n* = 8 per group, *p < 0.05 vs. BCP + vehicle group; ^#^ *p* < 0.05 vs. BCP + T10 3 μg/kg group

To assess whether T10 could ameliorate the BCP, different doses (1.5, 3, 12, 48, and 96 μg/kg) of T10 were i.t. injected once per day for 7 days from postoperative days (POD) 7 to 13 (Fig. 3a). Compared with BCP + veh group, i.t. administration of T10 significantly elevated the PWTs of the BCP rats in a dose-dependent manner (Fig. 3a–d), which effect started at POD 9, 2 days after the beginning of T10 treatment (Fig. 3a). The analgesic effects of T10 at 12, 48, and 96 μg/kg were still observed at POD 19, 6 days after the treatment stopped. However, the effect of T10 at lower dosages, 1.5 and 3 μg/kg, only lasted for 3–4 days after drug withdrawal. As summarized in AUC values of PWTs, the analgesia of T10 presented a significant group difference among the five dose regimes (Fig. 3b, *p* < 0.05). The effects of T10 were further calculated based on the log (dose)-response curve (Fig. 3d) that was calculated from the dose-response curve (Fig. 3c). The ED_50_ of T10 on bone cancer-induced mechanical allodynia was 5.874 μg/kg. Thus, the dose of 5 μg/kg, which was similar to the ED_50_ of T10, was chosen for subsequent experiments on mechanisms.

### i.t. T10 administration inhibited the bone cancer-induced glial activation in the spinal dorsal horn

**Fig. 4:**
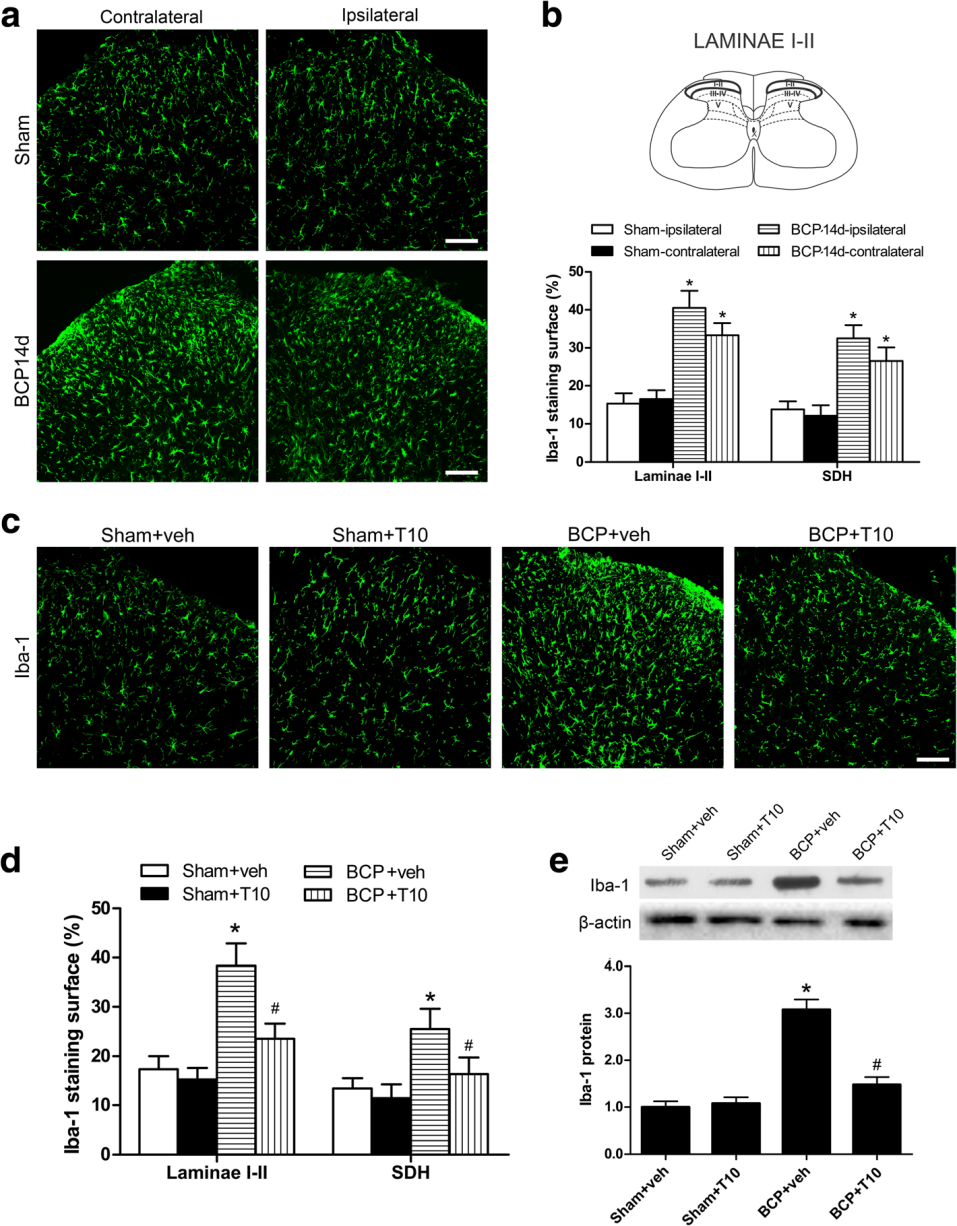
Effect of intrathecal administration T10 on microglial activation in the spinal dorsal horn (SDH) of BCP rats on POD 14. **a** Immunostaining of spinal sections showed the BCP-induced change of microglia marker, Iba-1, in the SDH. **b** Quantitative analysis of the percentage of Iba-1 immunostaining surface in the SDH showed the BCP-induced change (*n* = 4). **c** Immunostaining of Iba-1 in the ipsilateral SDH. **d** The percentage of Iba-1 immunostaining surface in the laminae I–II and whole SDH on the ipsilateral side. **e** Western blot analysis of the protein level of Iba-1 in the ipsilateral SDH, relative to that of sham + vehicle group (*n* = 5). Scale bar 100 μm. *p < 0.05 vs. sham + vehicle group; ^#^ *p* < 0.05 vs. BCP + vehicle group

**Fig. 5:**
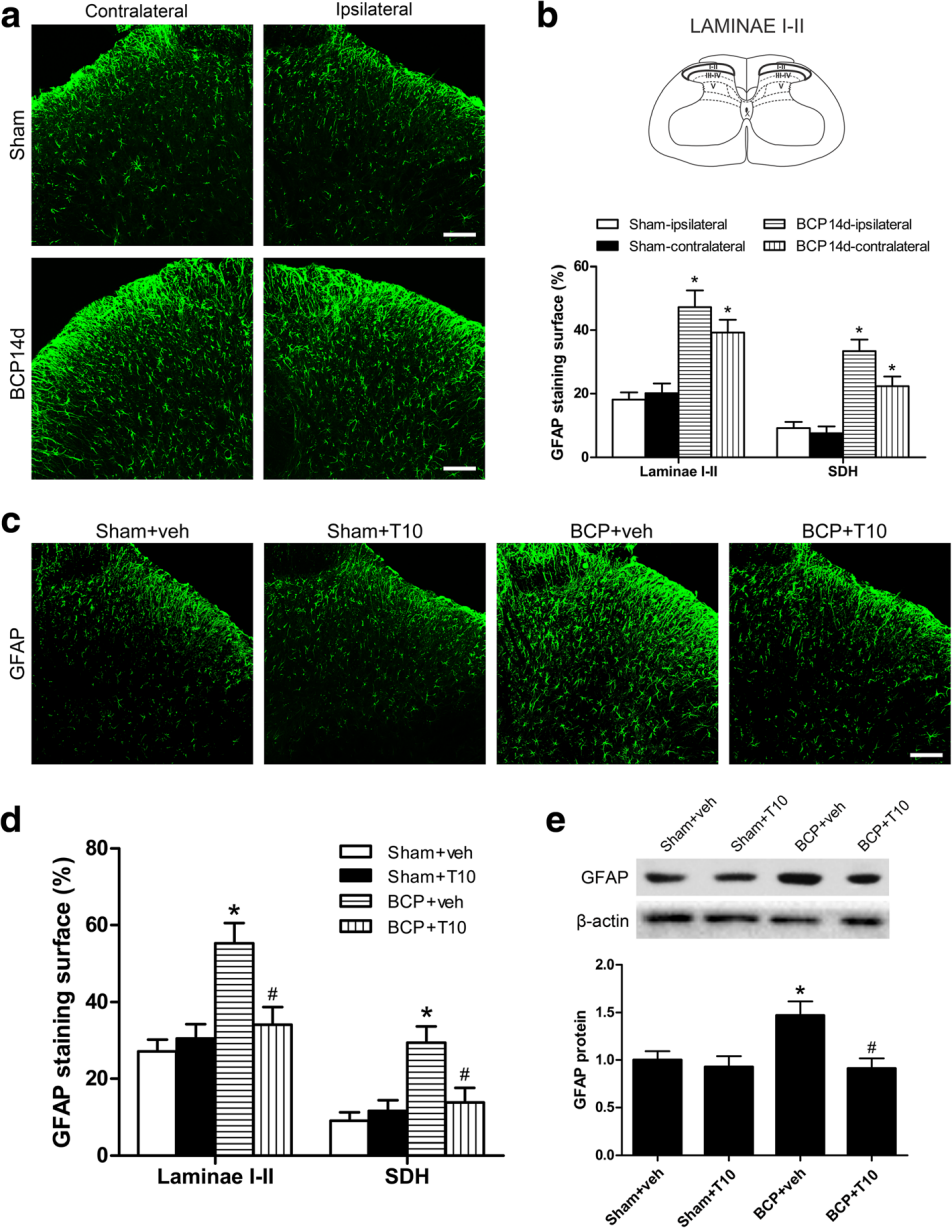
Effect of intrathecal T10 administration on astrocyte activation in the spinal dorsal horn (SDH) of BCP rats on POD 14. **a** Immunostaining of spinal sections showed the BCP-induced change of astrocyte marker, GFAP, in the SDH. **b** Quantitative analysis of the percentage of GFAP immunostaining surface in spinal cord laminae I–II and the whole SDH which showed the BCP-induced change (*n* = 4). **c** Immunostaining of GFAP in the ipsilateral SDH. **d** The percentage of GFAP immunostaining surface in the laminae I–II and whole SDH on the ipsilateral side. **e** Western blot analysis of the protein level of GFAP in the ipsilateral SDH, relative to sham + vehicle group (*n* = 5). Scale bar 100 μm. *p < 0.05 vs. sham + vehicle group; ^#^ *p* < 0.05 vs. BCP + vehicle group

We investigated the expression of Iba-1 (microglia marker) and GFAP (astrocyte marker) on POD 14 in various groups to identify whether the analgesic effect of T10 was mediated by its influence on the glial cells in spinal cord (Figs. 4 and 5). In immunofluorescent staining analysis, the staining surface percentages of Iba-1 and GFAP were calculated in the whole SDH and also in the spinal cord laminae I–II which is the main region involved in pain process in the SDH. The results showed significant enhancement of Iba-1 (Fig. 4a, b) and GFAP (Fig. 5a, b) expression in the bilateral SDH on POD 14, especially in the spinal cord laminae I–II, of BCP rats. The immunofluorescent data were further confirmed in Western blot analysis (Figs. 4e and 5e, BCP + veh group vs. sham + veh group, *p* < 0.05). However, daily intrathecal administration of T10 from POD 7 to 13 resulted in significantly decreased expression of both Iba-1 and GFAP in the SDH (Figs. 4c–e and 5c–e).

### i.t. T10 administration inhibited bone cancer-induced activation of MAPK pathways in the spinal dorsal horn

**Fig. 6:**
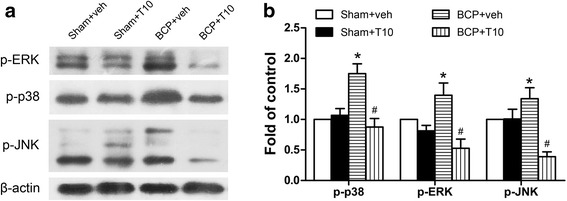
Effect of bone cancer inoculation and intrathecal T10 administration on MAPK pathways in the spinal dorsal horn (SDH) on POD 14. Western blot analysis of the protein level of p-extracellular signal-regulated kinase (ERK) and p-p38 and p-Jun N-terminal kinases (JNK) in the ipsilateral SDH, relative to sham + vehicle group (*n* = 4). Protein blot bands (**a**) and data analysis (**b**). *p < 0.05 vs. sham + vehicle group; ^#^ *p* < 0.05 vs. BCP + vehicle group

Since MAPK pathways have been shown to be involved in the glial activation and neuroinflammation in the spinal cord under several pathological pain conditions [30], we tested the levels of the three members of MAPKs, p-ERK, p-p38, and p-JNK, in the SDH of different groups on POD 14. Western blot analysis (Fig. 6) showed that bone cancer led to significant upregulation of all the three phosphorylated MAPKs (activated forms). After i.t. injection of T10, this upregulation of MAPKs pathways were reversed, while T10 treatment only made slight influence on the protein levels of p-ERK, p-p38, and p-JNK in sham group.

### Bone cancer induced increase of HDAC1 and HDAC2 in the SDH

**Fig. 7:**
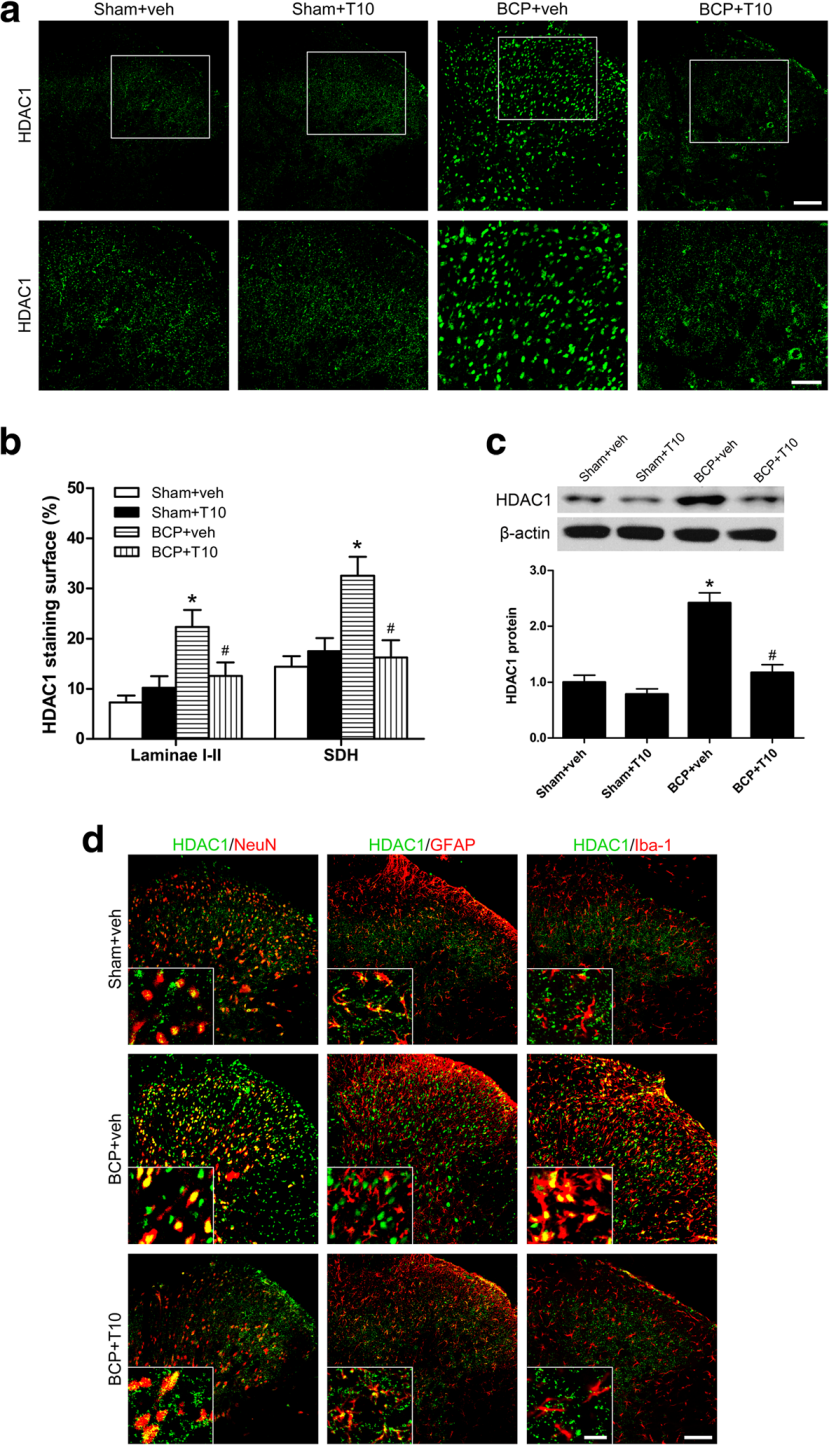
Effect of bone cancer inoculation and intrathecal T10 administration on HDAC1 in the spinal dorsal horn (SDH) on POD 14. **a** Ipsilateral SDH of the spinal sections showing the HDAC1 immunoreactive staining. The second row is the higher magnification images indicated in the white boxes in the first row. Scale bar 100 μm in the first row and 50 μm in the second row. **b** The percentage of HDAC1 staining surface in the laminae I–II and whole SDH on the ipsilateral side (*n* = 4). **c** Western blot analysis of the expression of HDAC1 in the SDH, relative to that of sham + vehicle group (*n* = 5). **d** Double-staining immunofluorescent images showing the distribution of HDAC1 (green) in neurons (NeuN, red), astrocytes (GFAP, red), and microglia (Iba-1, red) in different groups. Images in the white boxes are the amplification of an area in the corresponding image. Scale bar 100 μm outside of the white frame and 20 μm in the white frame. *p < 0.05 vs. sham + vehicle group; ^#^ *p* < 0.05 vs. BCP + vehicle group

**Fig. 8:**
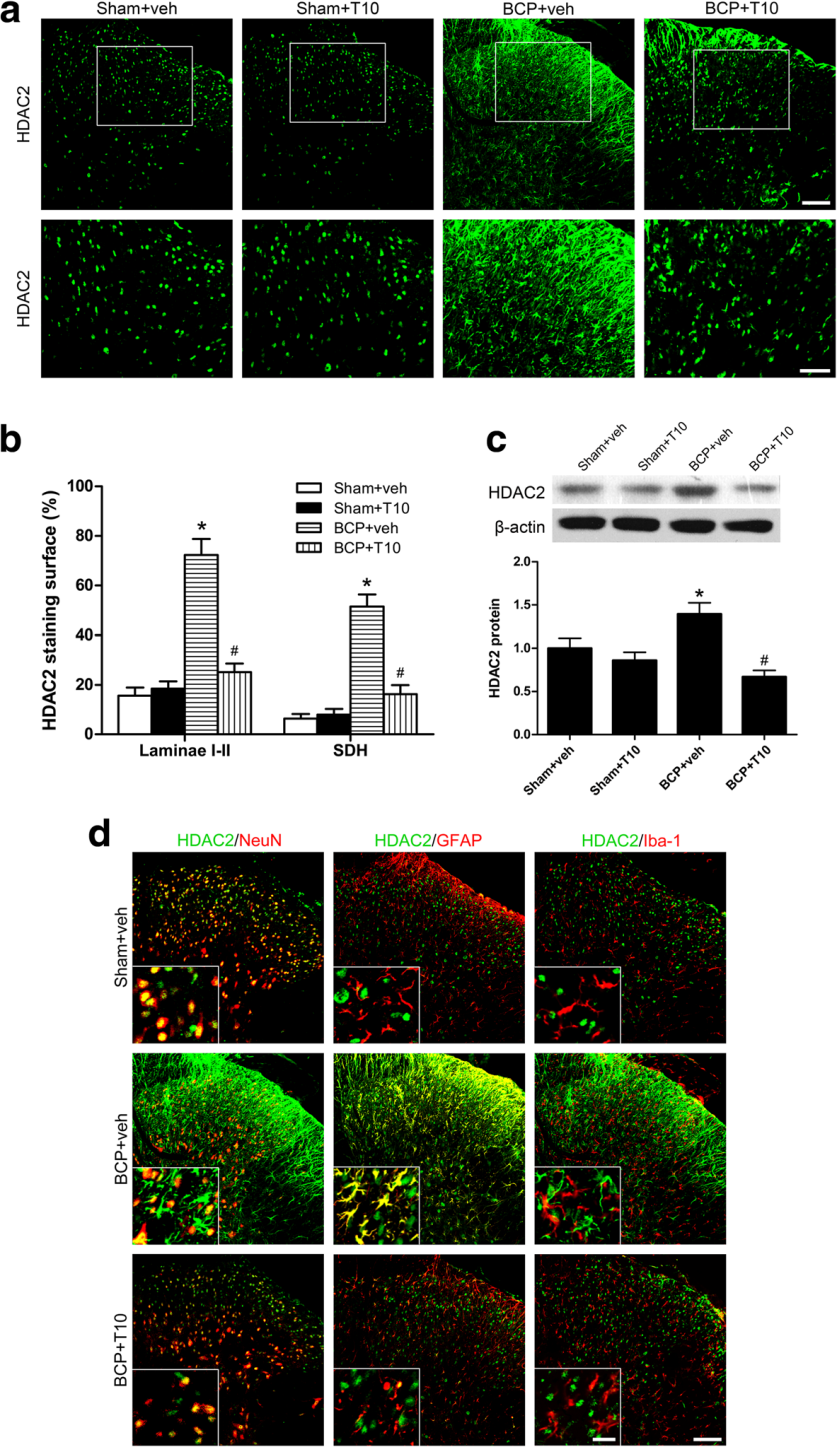
Effect of bone cancer inoculation and intrathecal T10 administration on HDAC2 in the spinal dorsal horn (SDH) on POD 14. **a** Ipsilateral SDH of the spinal sections showing the HDAC2 immunoreactive staining. The second row is the higher magnification images indicated in the white boxes in the first row. Scale bar 100 μm on the above and 50 μm on the below. **b** The percentage of HDAC2 staining surface in the laminae I–II and whole SDH on the ipsilateral side (*n* = 4). **c** Western blot analysis of the expression of HDAC2 in the SDH, relative to that of sham + vehicle group (*n* = 5). **d** Double-staining immunofluorescent images showing the distribution of HDAC2 (green) in neurons (NeuN, red), astrocytes (GFAP, red), and microglia (Iba-1, red) in different groups. Images in the white boxes are the amplification of an area in the corresponding image. Scale bar 100 μm outside of the white frame and 20 μm in the white frame. *p < 0.05 vs. sham + vehicle group; ^#^ *p* < 0.05 vs. BCP + vehicle group

To examine whether the analgesic effects of T10 and its influence on glia are related to epigenetic mechanisms, we examined the expression levels and cell distribution of HDAC1 and HDAC2 in the SDH on POD 14. Immunofluorescent staining showed that the distributions of HDAC1- and 2-like immunoreactivities were observed in the SDH. In comparison with sham + veh group, BCP + veh group showed increased expressions of both HDAC1 (Fig. 7a, b) and 2 (Fig. 8a, b), especially in laminae I–II which regions in the spinal cord are closely involved in pain transduction. In the ipsilateral SDH, Western blot analysis further showed that bone cancer induced significant increase of the protein levels of both HDAC1 and HDAC2 in the SDH at POD 14 (Figs. 7c and 8c). Remarkably, immunofluorescent double-staining showed the specific cell-type of the changes of these two HDACs. In the SDH of sham + veh rats, HDAC1 is mainly expressed in astrocytes and a few in neurons or microglia (Fig. 7d). However, 14 days after BCP surgery, HDAC1 was sharply increased in the nucleus of both neurons and microglia but decreased in astrocytes in the SDH (BCP + veh group, Fig. 7d). In addition, HDAC2 is mainly expressed in the nucleus of neurons in the SDH of sham + veh group but obviously increased in astrocytes in BCP + veh group on POD 14 (Fig. 8d).

### T10 treatment inhibited the bone cancer-induced increase of HDAC1 and HDAC2 in the SDH

On POD 14, compared with BCP + veh group, the levels of HDAC1 and HDAC2 in the SDH of BCP + T10 group were significantly decreased (Figs. 7 and 8), especially in the glial cells (Figs. 7d and 8d). In addition, compared with sham + veh group, the expressions of HDAC1 and HDAC2 in sham + T10 group were not obviously influenced by T10 treatment (Figs. 7c and 8c).

### HDACs and astrocytes in the SDH were re-upregulated after the withdrawal of low doses of T10

**Fig. 9:**
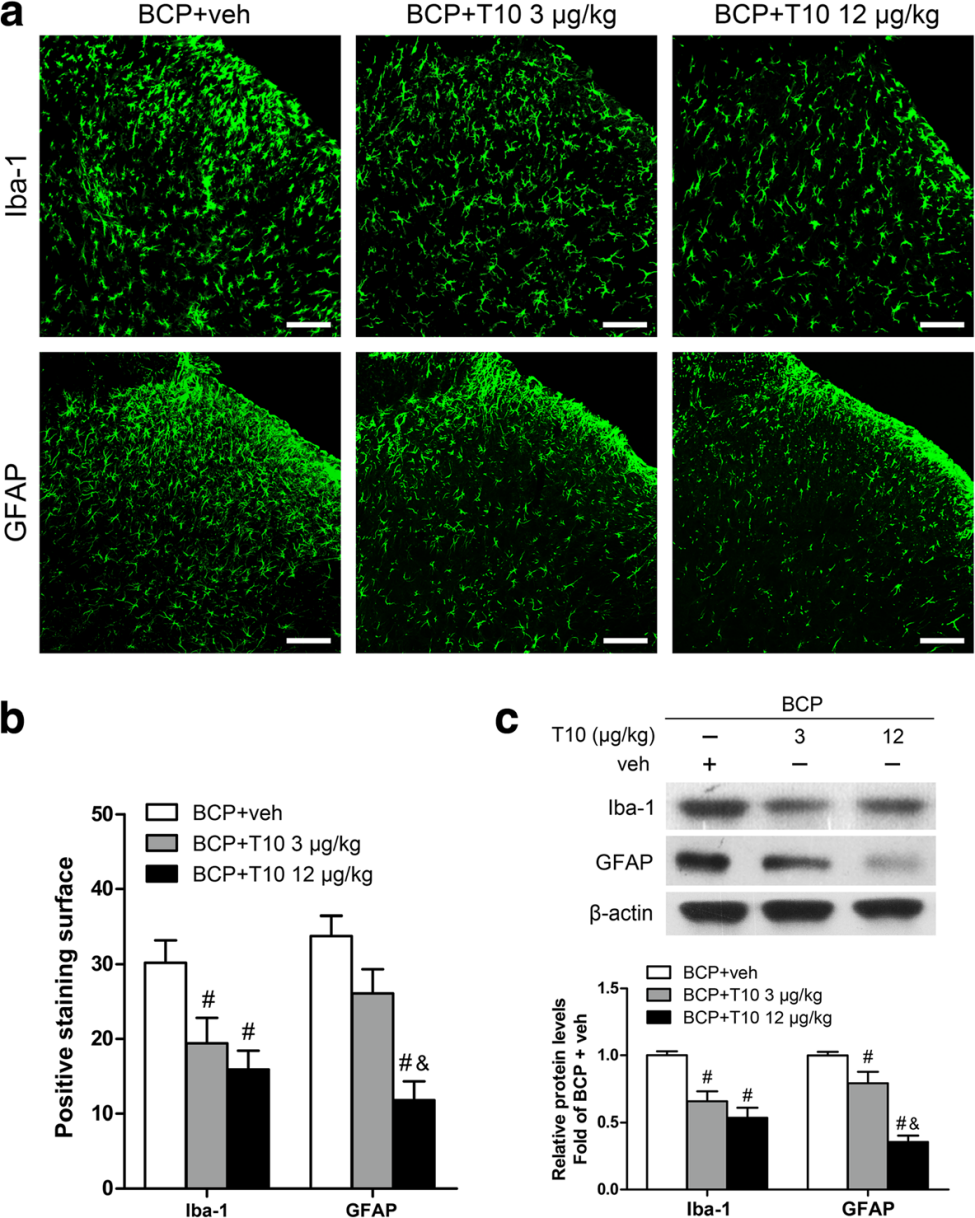
Effect of intrathecal T10 administration on microglia and astrocytes in the SDH on POD 19. **a** Ipsilateral SDH of the spinal sections showing the Iba-1 and GFAP immunoreactive staining. Scale bar 100 μm. **b** The percentage of Iba-1 and GFAP staining surface in the ipsilateral SDH (*n* = 3). **c** Western blot analysis of the expressions of Iba-1 and GFAP in the ipsilateral SDH, relative to that of BCP + vehicle group (*n* = 4). ^#^ *p* < 0.05 vs. BCP + vehicle group; ^&^ *p* < 0.05 vs. BCP + T10 3 μg/kg group

**Fig. 10:**
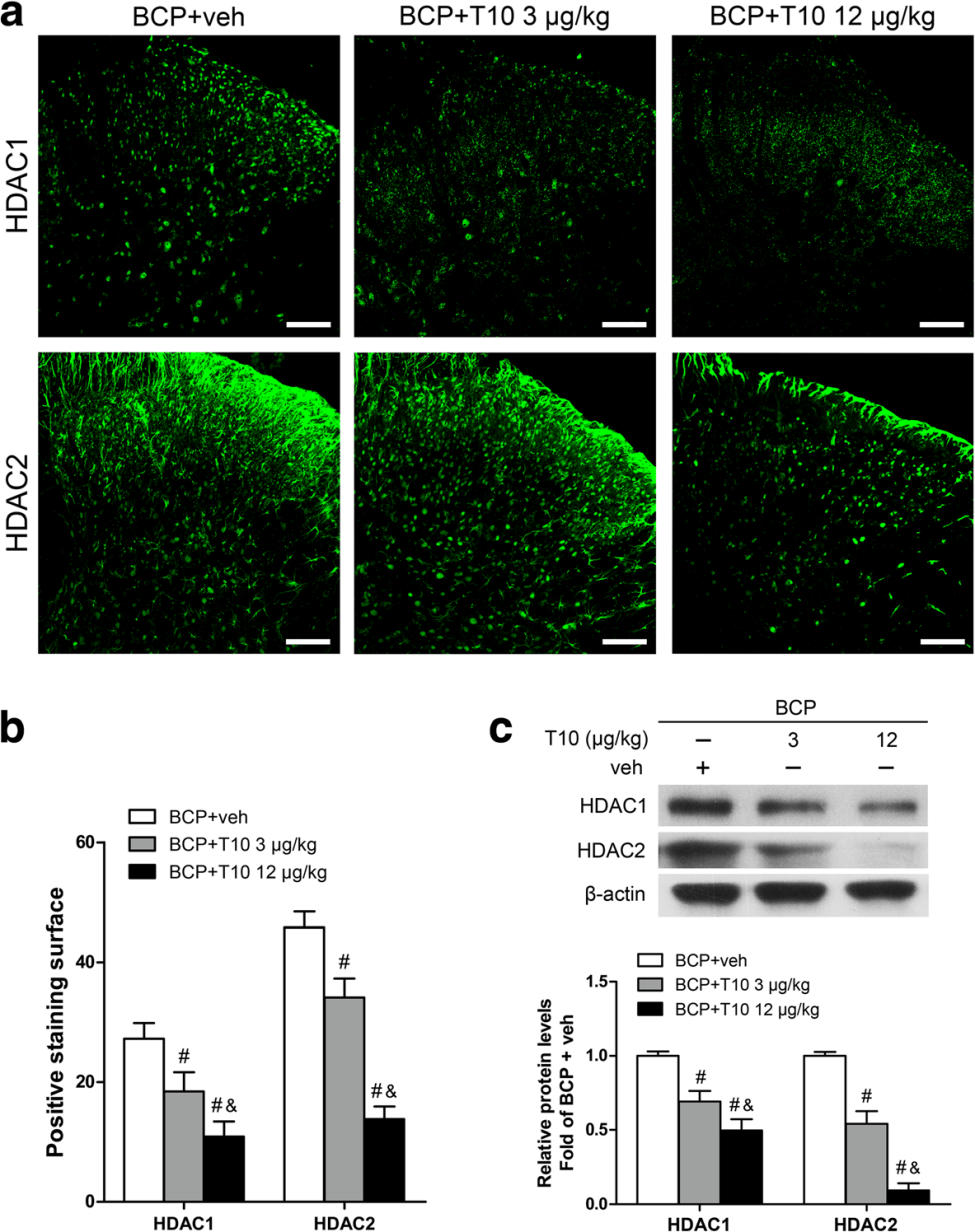
Effect of intrathecal T10 administration on HDAC1 and HDAC2 in the SDH on POD 19. **a** Ipsilateral SDH of the spinal sections showing the HDAC1 and HDAC2 immunoreactive staining. Scale bar 100 μm. **b** The percentage of HDAC1 and HDAC2 staining surface in the ipsilateral SDH (*n* = 3). **c** Western blot analysis of the expressions of HDAC1 and HDAC2 in the ipsilateral SDH, relative to that of BCP + vehicle group (*n* = 4). ^#^ *p* < 0.05 vs. BCP + vehicle group; ^&^ *p* < 0.05 vs. BCP + T10 3 μg/kg group

To investigate why the mechanical allodynia of BCP rats treated with relatively lower doses of T10, i.e., 1.5 and 3 μg/kg, were gradually recovered after treatment was stopped, and why the analgesic effects of T10 at doses higher than 12 μg/kg were still observed on POD 19, 6 days after drug withdrawal, we further examined the HDACs and glial cells in the SDH of BCP animals on POD 19 (Figs. 9 and 10). Compared with BCP + veh group, BCP + T10 12 μg/kg group still showed significantly lower levels of HDAC1 and 2 as well as glial cell markers (Iba-1 and GFAP) on POD 19. This was consistent with the reversed analgesic effects of T10 in BCP + T10 12 μg/kg group after the drug was stopped (Fig. 3a). BCP + T10 3 μg/kg group, however, showed significantly higher levels of HDACs and GFAP than those of BCP + T10 12 μg/kg group, which might be associated with the recovery of mechanical allodynia in the BCP rats treated with lower doses of T10. The difference of HDAC2 levels between these two T10-treated groups was more obvious than that of HDAC1, which might be linked to the re-activation of astrocytes on POD 19 because HDAC2 was expressed in astrocytes and neurons but not in microglia (Fig. 8d).

## Discussion

Currently, the management of BCP, which severely influence the quality of life of cancer patients with bone metastasis, remains far from satisfactory. The present study was to examine the antinociceptive effect and possible mechanism of triptolide (T10) in the BCP rat model. Our major findings were as follows: First, the long-lasting antinociceptive effect of T10 was observed in the BCP rats in a dose-dependent manner, whose effect lasted for several days after the drug was stopped. Second, bone cancer induced bilateral activation of microglia and astrocytes in the SDH, while intrathecal injection of T10 reversed the glial activation. Third, bone cancer induced significant upregulation of MAPK signaling pathways and expression increase of HDAC1 in the microglia and neurons in the SDH as well as remarkable increase of HDAC2 in astrocytes, which were effectively inhibited by T10 treatment. Fourth, after lower doses of T10 treatment were stopped, the HDAC1 and 2 were re-upregulated accompanied by the reactivation of astrocytes in the SDH and the gradual re-occurrence of mechanical allodynia of the hind paws. These results suggest that T10 may exert epigenetic regulation activity, thus inhibiting the pathological activation of glial cells in the SDH and the consequent BCP.

The present study showed that intrathecal administration of T10 inhibited bone cancer-induced allodynia in a dose-dependent manner, which is in line with a previous report showing the analgesic effect of T10 on the chronic pain in a rat model of bone cancer pain [17]. Interestingly, after drug withdrawal, the analgesic effect of T10 at relatively higher doses was still maintained till 6 days later. Our study used intrathecal injection to focus the effects of T10 on the nervous system at spinal level in BCP conditions. Growing evidence, however, has shown that T10 exerts considerable anti-tumor activities on various cancers [6]. Some T10 analogs, such as Minnelide (a more water-soluble analog of T10), is currently in phase I clinical trials to treat pancreatic cancer [31, 32]. These long-term analgesic and anti-tumor actions may make T10 a potential therapeutic agent for BCP. In another experiment of our group, when used in combination with morphine, T10 not only improved the analgesic effects of morphine on BCP, but also attenuated the morphine tolerance (unpublished data). Therefore, in the BCP treatment, T10 could possibly be used in combination with relatively lower doses of current analgesics, such as morphine, and anti-tumor drugs, such as chemotherapy agents. This drug combination could not only achieve better management of both pain and bone cancer but also reduce the side effects of long-term morphine or anti-tumor treatment, which need further investigation in the future.

Reciprocal signaling between immunocompetent cells in the central nervous system (CNS), mainly microglia and astrocytes, has emerged as a key phenomenon underpinning pathological and chronic pain mechanisms [18, 19]. In the present study, bone cancer induced by intratibial inoculation of Walker 256 mammary gland carcinoma cells led to the activation of microglia and astrocytes in the SDH, which is in line with previous studies [20–23]. Several mechanisms may account for this glial activation in BCP conditions. First, bone tumor could stimulate and even damage the nerves innervating the affected bone, while periphery nerve injury could induce sensitization of primary sensory neurons and abnormal release of neurotransmitters and mediators, such as fractalkine, from their central process, leading to the activation of glial cells in the SDH [3, 33]. Second, bone cancer could induce proinflammatory cytokines secretion from several cells in the tumor microenvironment, such as tumor cells, tumor-injured sensory neurons, and tumor-induced osteoclasts [3, 34], while studies in neuro-immune interaction have demonstrated that inflammatory mediators from peripheral inflammation contribute to the pathological alterations of glial cells in the CNS [18]. Recent studies have reported that, in bone cancer, chemokines and their receptors, such as CXCL12/CXCR4 [22], CCL2 [35], and CXCR3 [36], mediate the activation of microglia and astrocytes in the SDH via intracellular signals including MAPKs and NF-κB pathways, consistent with our results that MAPK pathways were activated in the SDH by bone cancer. These pathologically activated glia can further secrete various neuro and glial excitatory substances including CCL1 and CXCL12, leading to not only neuronal sensitization, i.e., enhanced neural excitability facilitating pain processing, but also more glial proliferation and activation, which is implicated in bone cancer pain [3, 33].

Interestingly, our results showed that glial activation appeared in both ipsilateral and contralateral SDH in BCP rats on POD 14. This was consistent with the bilateral mechanical allodynia of hind paws in BCP rats. Such pain behaviors on the contralateral side, which are called “mirror-image pain,” have been observed in both human and animal studies including BCP model [37] and also non-cancer pain status [38–40]. Although the underlying mechanism still not totally clear, it has been indicated that the neuroimmune responses, mainly the activation of astrocytes and microglia as well as the consequent neuroinflammation in the contralateral dorsal spinal cord, play an important role in the occurrence of mirror-image pain. The issue seems confused by the different results in previous studies about microglia and astrocytes in mirror-image pain, and it has been proposed that the type of glia that is activated might rely on the original pain cause [40]. In the present study, both microglia and astrocytes were activated in the contralateral spinal cord of BCP rats, which is in line with the report form Mao et al. [37]. These results suggested that neuroimmune response in the contralateral SDH might be implicated in the pain behaviors of contralateral hind paw under BCP conditions. By now, it is still elusive what lead to the contralateral spinal glial activation and how the glia transfer pain message to the contralateral side. Some possible mechanisms have been supposed as the following [40]: (i) the Ca^2+^ wave and astroglial Ca^2+^ transients or oscillations may spread within the astroglial networks and then promote new synapse connection, (ii) the primary activated glia release proinflammatory cytokines which serve as a secondary stimulus to activate the glia consistently, (iii) the glia are activated by the pain messages from neurons transmitted from the ipsilateral spinal cord in time, including substance P (SP) and calcitonin gene-related peptide (CGRP) which are paracrined and can slowly spread within the spinal cord to activate the glia. However, the exact mechanism underlying the contralateral spinal glial activation and the mirror-image pain under BCP conditions still need further investigation.

It has been reported that, in neuropathic pain model, T10 could inhibit the spinal glial activation and the consequent release of proinflammatory and neurotoxic factors, thus suppressing the upregulation of neuronal NR2B expression and chronic pain behaviors [15, 16]. In the present study, T10 treatment significantly inhibited the BCP-induced activation of microglia and astrocytes in the SDH, accompanying the amelioration of pain behaviors. This is consistent with previous studies indicating that T10 has strong anti-inflammatory activities on microglia and astrocytes in other chronic pain models [15, 16]. Recent studies also indicated that T10 could attenuate Parkinson’ disease and Alzheimer’s disease via inhibiting glial activation-mediated neuroinflammation and protecting neurons from inflammatory insults [5, 41, 42]. All these results indicated that the analgesic effect of T10 on BCP is related to its anti-inflammatory action on glial activation. Then, there comes the question how T10 could influence glial cells, which is also a common question in the field of mechanisms underlying the effects of T10 in the CNS.

Although T10 has been attracting worldwide attention because of a wide range of medicinal value, such as anti-inflammatory, anti-tumor, neuroprotective, and cardiovascular effects, the receptor of T10 in cells and the exact molecular mechanism underlying these effects are still unclear [5]. MAPK family, including ERK, p38, and JNK, serve as important signals in mediating many inflammatory responses [43]. It has been shown that MAPK activation is implicated in the glial activation in many diseases in the CNS related to neuroinflammation [44], such as Parkinson’ disease [45] and several pathological pain including BCP [30]. In animal studies on BCP, the activation of MAPKs in the spinal cord has been shown to contribute to the activation and inflammatory mediator release of glia as well as the chronic pain status [30]. On the other hand, the modulation of MAPKs has been shown to be closely related to the inhibitory effects of T10 on the spinal glial activation in neuropathic pain [15]. In the present study, we first reported the inhibitory effects of T10 on the BCP-induced activation of MAPKs in the spinal cord, which might be associated with the inhibition of glial activation. But still, how does T10 modulate these intracellular signal molecules, like MAPKs, remain far from clear.

Increasing evidence shows that epigenetics is involved in gene expression and synaptic plasticity of chronic pain states [24]. Recently, it has been attracting more and more attention that epigenetic mechanisms play an indispensable role in the regulation of glia [46] and, specifically, in the control of microglial activation during neuroinflammation [47]. HDAC inhibition treatment has been shown to be beneficial for chronic pain, neurodegenerative diseases and CNS injury, which have been linked to the inhibition of glia-related neuroinflammation [47]. The present study showed that tumor cell inoculation in tibia induced significantly increased expression of HDAC1 in the microglia and neurons in the SDH, with HDAC2 increased markedly in astrocytes. The localization alteration of HDACs in the glial cells showed a “from nothing to high” pattern. Previous studies have shown that treatment with HDAC inhibitors, such as scriptaid, SAHA, VPA [48], and TSA [49], inhibited microglial activation and led to a decrease in inflammatory markers. In another concurrent study of our lab, treatment with an HDAC inhibitor, SAHA, significantly reversed the BCP-induced upregulation of HDACs in the SDH, thus inhibited the activation of spinal glial cells and the BCP behaviors (unpublished data). In the studies on the anti-tumor effects of T10, it has been demonstrated that T10 can regulate histone modifications by altering molecules like histone methyltransferases and demethylases [25, 26]. However, it still remains unclear whether T10 helps to ameliorate diseases in the nervous system via epigenetic mechanisms. Our results showed that intrathecal T10 reduced the protein levels of HDAC1 and 2 in the SDH of BCP rats on POD 14, especially in microglia and astrocytes, to a level comparable to that of sham control group, which is accompanied by the attenuation of both glial activation and pain behaviors.

After drug stop, although the analgesic effects of T10 were reserved for several days, the effects gradually fade down and the pain behaviors of BCP rats treated with lower doses of T10, namely, 1.5 or 3 μg/kg, appeared again 6 days after drug withdrawal. This suggested that some changes might happen in the nervous system after drug stop. Our further experiments showed that, on POD 19, although the glia markers and HDACs were still at low levels in BCP + 12 μg/kg group, HDACs and GFAP were re-upregulated in BCP + T10 3 μg/kg group, which might be respectively related to the reserved and disappeared analgesic effects of T10 in these two groups. Between the two HDACs, the more conspicuous re-upregulation of HDAC2 might be associated with the re-activation of astrocytes, because HDAC2 was expressed in astrocytes and neurons but not in microglia (Fig. 7d). These results above together indicate that HDAC upregulation may contribute to the bone cancer-induced activation of glial cells in the SDH and that the regulation of glial cells by T10 is, at least partially, through epigenetic mechanisms for histone acetylation.

In the present experiment, the analgesic effects of T10 at relatively higher doses were still observed within 6 days after the drug was stopped. It is known that epigenetic marks are typically more stable than the rapidly modulated post-translational modifications of signaling proteins and may persist after the original stimulus has resolved [50]. Epigenetics provides a mechanism for converting transient short-lived signals into more persistent cellular responses lasting several hours or days (or longer) [51]. This may account for the sustained analgesic effect of T10 after drug stop. Further studies are needed to investigate the exact mechanisms of the interaction between T10 and epigenetic elements, such as HDACs.

## Conclusions

The present study demonstrated that pathological activation of microglia and astrocytes in spinal dorsal horn associated with HDACs increase contribute to bone cancer pain (BCP). T10 reversed the glial activation, possibly by inhibiting the upregulation of HDACs, and ameliorated the chronic pain. Our findings provide new insights into the mechanisms of the pharmacological effects of T10 via epigenetic modulation in glial cells and suggest T10 as a novel promising drug for the treatment of cancer pain, especially for BCP.

## Abbreviations

BCP: Bone cancer pain
GFAP: Glial fibrillary acidic protein
HDAC: Histone deacetylase
Iba-1: Ionized calcium-binding adaptor molecule 1
PWTs: Paw withdrawal thresholds
SDH: Spinal dorsal horn
